# Cell-Penetrating Peptide Based on Myosin Phosphatase Target Subunit Sequence Mediates Myosin Phosphatase Activity

**DOI:** 10.3390/biom15050705

**Published:** 2025-05-12

**Authors:** Andrea Kiss, Mohamad Mahfood, Zsófia Bodogán, Zoltán Kónya, Bálint Bécsi, Ferenc Erdődi

**Affiliations:** Department of Medical Chemistry, Faculty of Medicine, University of Debrecen, H-4032 Debrecen, Hungary; mahfood.mohamad@med.unideb.hu (M.M.); bodogan.zsofia@med.unideb.hu (Z.B.); konya.zoltan@med.unideb.hu (Z.K.); bbalint@med.unideb.hu (B.B.); erdodi@med.unideb.hu (F.E.)

**Keywords:** myosin phosphatase, protein phosphatase-1 (PP1) catalytic subunit (PP1c), myosin phosphatase target subunit-1 (MYPT1), cell-penetrating MYPT1 peptide, transactivating transcriptional activator (TAT)-peptide

## Abstract

Myosin phosphatase (MP) holoenzyme consists of protein phosphatase-1 (PP1) catalytic subunit (PP1c) associated with myosin phosphatase target subunit-1 (MYPT1) and it plays an important role in mediating the phosphorylation of the 20 kDa light chain (MLC20) of myosin, thereby regulating cell contractility. The association of MYPT1 with PP1c increases the phosphatase activity toward myosin; therefore, disrupting/dissociating this interaction may result in inhibition of the dephosphorylation of myosin. In this study, we probed how MYPT1^32–58^ peptide including major PP1c interactive regions coupled with biotin and cell-penetrating TAT sequence (biotin-TAT-MYPT1) may influence MP activity. Biotin-TAT-MYPT1 inhibited the activity of MP holoenzyme and affinity chromatography as well as surface plasmon resonance (SPR) binding studies established its stable association with PP1c. Biotin-TAT-MYPT1 competed for binding to PP1c with immobilized GST-MYPT1 in SPR assays and it partially relieved PP1c inhibition by thiophosphorylated (on Thr696 and Thr853) MYPT1. Moreover, biotin-TAT-MYPT1 dissociated PP1c from immunoprecipitated PP1c-MYPT1 complex implying its holoenzyme disrupting ability. Biotin-TAT-MYPT1 penetrated into A7r5 smooth muscle cells localized in the cytoplasm and nucleus and exerted inhibition on MP with a parallel increase in MLC20 phosphorylation. Our results imply that the biotin-TAT-MYPT1 peptide may serve as a specific MP regulatory cell-penetrating peptide as well as possibly being applicable to further development for pharmacological interventions.

## 1. Introduction

The phosphorylation of the 20 kDa light chain of myosin II (MLC20) plays an essential role in the regulation of contractility of smooth muscle as well as several other cell lines [1]. The extent of MLC20 phosphorylation is balanced by the phosphorylating protein kinases; for example, myosin light chain kinase (MLCK), RhoA activated kinase (ROK), and zipper activated kinase (ZIPK), and the dephosphorylating myosin phosphatase (MP). Although the regulatory role of kinases in MLC20 phosphorylation has not been debated, nevertheless, for the past few decades, the focus has been on the involvement of MP in this process, and the emphasis has been on its role in the physiological Ca^2+^-sensitization of smooth muscle contraction [2].

MP belongs to the phosphoserine/threonine (P-Ser/Thr) specific protein phosphatase-1 (PP1) family and the trimer MP holoenzyme includes the δ/β isoform of PP1 catalytic subunit (PP1cδ/β, termed PP1c further on), a 130–133 kDa myosin phosphatase target subunit (MYPT) and a 20 kDa protein associating with MYPT with a yet unclear function in MP activity [3,4,5]. Many MYPT isoforms exist; however, MYPT1 is the one ubiquitously expressed in many cells. MYPT1 has important roles in the regulation of MP activity. It binds to both PP1c and myosin, thereby targeting the substrate to the catalytic site of PP1c. Because of these interactions, MYPT1 greatly increases the activity of PP1c toward P-myosin as well as isolated P-MLC20 substrates [6,7]. In contrast, interactions with MYPT1 decreases PP1c activity toward phosphorylase substrate. On the other hand, phosphorylation of MYPT1 on Thr696 and Thr853 results in inhibition of MP activity [8]. Several kinases may catalyze the phosphorylation of these residues and it represents a major mechanism in the physiological regulation of MP [4]. Another cellular mechanism for inhibition of MP is phosphorylation of C-kinase activated phosphatase inhibitor of 17 kDa (CPI-17) which binds to both PP1c and MYPT1 suppressing the dephosphorylation of P-myosin [9].

PP1c isoforms associate with many distinct regulatory and/or substrate proteins in the cells and it has been shown that these interactions are governed in most of the cases by a consensus binding motif of RVxF sequence in the interacting proteins [10]. In the regulatory proteins the presence of the RVxF sequence is essential to stable interaction with PP1c. This recognition led to the idea to apply RVxF sequence containing short cell-penetrating peptides to disrupt interactions between PP1c and its binding partners [11,12,13]. Thorough analyses of the influence of these disrupting peptides have proven to be specific toward PP1 holoenzymes with diverse effects that include increase or decrease of the dephosphorylation of several PP1 designated substrates [14]. However, besides the PP1 specificity, these peptides did not differentiate between the various PP1 forms in which PP1c associated with distinct regulatory or inhibitor proteins.

In the MP holoenzyme, MYPT1 also includes an RVxF binding motif (^35^KVKF^38^) and it was shown that while this sequence was essential for interaction with PP1c other regions N-terminal (Myosin Phosphatase N-terminal Element, defined as MyPhoNE) or C-terminal (ankyrin repeats) to the ^35^KVKF^38^ sequence were also involved in stabilizing the PP1c-MYPT1 complex [6]. In the present work, we have planned a cell-penetrating peptide that includes the RVxF motif in MYPT1 complemented with a peptide segment (part of the first ankyrin repeat) including key interacting residues with PP1c [15] for possible disruption of MP holoenzyme. Our results indicate the capability of this peptide to disrupt PP1c-MYPT1 interaction, and thereby its mediation in myosin phosphatase activity in vitro and in cells.

## 2. Materials and Methods

### 2.1. Peptides and Proteins

Biotin-TAT and biotin-TAT-MYPT1 peptides were from AnaSpec (San Jose, CA, USA). Turkey gizzard myosin [16] and the 20 kDa myosin light chain (MLC20) [6] were purified and phosphorylated using [γ^32^P]ATP (Hungarian Isotope Institute, Budapest, Hungary) as described previously. Catalytic subunit of protein phosphatase-1 (PP1c) [6] and myosin phosphatase (MP) holoenzyme [17] were purified from rabbit skeletal muscle and turkey gizzard, respectively. The δ isoform of PP1c (PP1cδ) was expressed in *E. coli* and purified with affinity chromatography [6]. GST-MYPT1 was expressed, purified and thiophosphorylated with Rho kinase in the presence of ATP-γ-S, as detailed earlier [18].

### 2.2. Phosphatase Assays

Phosphatase activities were determined with 1 μM ^32^P-labelled MLC20 (^32^P-MLC20) or myosin (^32^P-myosin) as substrates at 30 °C in 20 mM Tris-HCl (pH 7.4) and 0.1% 2-mercaptoethanol. Native MP holoenzyme (1 nM) was pre-incubated with 0, 0.1, 1, 5, 10 or 20 µM biotin-TAT/TAT-MYPT1 peptides for 10 min, and then the reaction was initiated by addition of the substrate. After 10 min incubation the reaction was terminated and the released ^32^P_i_ was determined as described earlier [6]. Native PP1c (1 nM) was pre-incubated in the absence or presence of 1 µM biotin-TAT/TAT-MYPT1 followed by 10 min incubation with 0, 1, 10 or 100 nM thiophosphorylated GST-MYPT1 before addition of ^32^P-MLC20 substrate.

Myosin phosphatase activity of minimally diluted A7r5 cell lysates was assayed with ^32^P-myosin for 1 min. Phosphatase activities were expressed as percentage of control (untreated sample).

### 2.3. Streptavidin Sepharose Affinity Chromatography

Streptavidin Sepharose (GE Healthcare, Chicago, IL, USA) was equilibrated with binding buffer (20 mM Tris pH 7.4, 0.15 M NaCl) and then biotinylated TAT or TAT-MYPT1 was captured to the column. After washing the column with five column volumes of binding buffer, purified PP1c was applied and allowed to bind. The column was washed with 10 column volumes of binding buffer, followed by elution with 10 column volumes of 20 mM Tris pH 7.4 containing 0.6 M NaCl and then with 10 column volumes of 20 mM Tris pH 7.4 containing 3 M KSCN. Phosphatase activity of the eluted fractions was assayed with ^32^P-MLC20 substrate as described above.

### 2.4. Surface Plasmon Resonance Binding Experiments

Surface plasmon resonance (SPR) experiments were performed on a Biacore 3000 instrument (Biacore AB, Uppsala, Sweden).

Sensor chip SA (Biacore AB) was conditioned with three consecutive one-minute injections of 1 M NaCl in 50 mM NaOH before the ligand was immobilized. Biotinylated TAT or TAT-MYPT1 peptides were injected over the sensor chip surfaces in a concentration of 2 μM for 5 min at a flow rate of 10 μL/min. Next, PP1cδ was injected over the surface at a concentration of 0.1 μM for 15 min and then the dissociation phase was monitored for 6 min. Binding of PP1cδ to the immobilized peptides was monitored as a sensorgram where response unit (RU) values were plotted against time.

Anti-GST antibody was immobilized on a XanTec sensor chip SCB CMD500L (XanTec Bioanalytics GmbH, Duesseldorf, Germany) by the amine-coupling method and then recombinant full-length GST-MYPT1 or GST were captured. Next, 3 μM recombinant PP1cδ was injected over the surfaces in the absence or presence of 1, 5 or 10 μM biotin-TAT-MYPT1 peptide or 10 μM biotin-TAT (as control) at a flow rate of 10 μL/min and the binding of PP1cδ was monitored as changes of the RU in time. The association phase of the interactions was monitored for 7 min and the dissociation phase in running buffer without the analytes was followed for 6 min. The control surface (GST) was treated identically to the ligand surfaces (GST-MYPT1) to determine unspecific binding, which was subtracted from the data obtained with the ligand surfaces.

Sensorgrams were evaluated using BIAevaluation 3.1 software (Biacore AB) and plotted with GraphPad Prism9 (GraphPad Software Boston, MA, USA).

### 2.5. Cell Cultures, Treatments and Transfections

TsA201 cells (from ECACC, Salisbury, UK) and A7r5 cells (from ATCC, Manassas, VA, USA) were cultured according to supplier’s recommendations.

Sub-confluent A7r5 cell cultures were incubated with 0, 1 or 10 µM biotin-TAT/TAT-MYPT1 peptide for 30 min. Whole cell lysates were prepared and analyzed by Western blotting using anti-MLC20^pS19^ (Cell Signaling, Danvers, MA, USA) and anti-actin (Sigma-Aldrich, St. Louis, MO, USA) antibodies. MLC20^pS19^ signals were normalized to actin and were expressed relative to the average of the untreated samples.

For phosphatase assay, cells were lysed in Tris-buffered saline containing 0.1 mM EDTA and 0.5% protease inhibitor cocktail (Sigma-Aldrich). Lysates were sonicated, centrifuged, and the supernatants were assayed as described above.

TsA201 cells were grown to 60–80% confluency and transfected with pReceiver-M11/Flag-MYPT1 plasmid (Genecopoeia, Rockville, MD, USA) using jetPEI (Polyplus, Illkirch, France) transfection reagent according to the manufacturer’s recommendations.

### 2.6. Immunofluorescent Staining

A7r5 cells were incubated with 10 µM biotin-TAT/TAT-MYPT1 peptide for 30 min. Cells were fixed with paraformaldehyde (4%) and permeabilized with 0.02% (*v*/*v*) Triton X-100. After blocking in 1% (*w*/*v*) sterile BSA in PBS, coverslips were incubated with Alexa Fluor 488-conjugated Streptavidin (Thermo Fisher Scientific, Waltham, MA USA) in 1% (*w*/*v*) BSA/PBS. For nuclear staining, propidium iodide (PI) was applied. Images were taken with a Zeiss Axiolab microscope (Carl Zeiss Inc., Thornwood, NY, USA).

### 2.7. Pull-Down Assays

TsA201 cells expressing Flag peptide-coupled MYPT1 (Flag-MYPT1) were lysed and Flag-MYPT1 together with its associated proteins were isolated on anti-Flag affinity gel (Sigma-Aldrich) according to the manufacturer’s instructions. The resin was incubated with 0, 1 or 10 µM biotin-TAT/TAT-MYPT1 peptide containing TBS for 15 min at room temperature. Then, the resin was washed with TBS and the resin-bound proteins were solubilized by boiling in SDS sample buffer and were subjected to Western blotting using anti-Flag (Sigma-Aldrich) and anti-PP1cδ (Merck, Darmstadt, Germany) antibodies. PP1cδ signals were normalized to Flag-MYPT1 signals and were expressed relative to the average of the untreated samples.

### 2.8. Statistical Analysis

Quantified data are presented as mean ± standard deviation (SD) of *n* = 3–5 experiments. Statistical analysis was performed using unpaired two-tailed *t*-test using GraphPad Prism9 software. A *p* value of less 0.05 was considered significant (*p* < 0.05 (*), *p* < 0.01 (**) and *p* < 0.001 (***)).

## 3. Results and Discussion

### 3.1. Design of the PP1c-MYPT1 Disrupting Peptide

**Figure 1:**
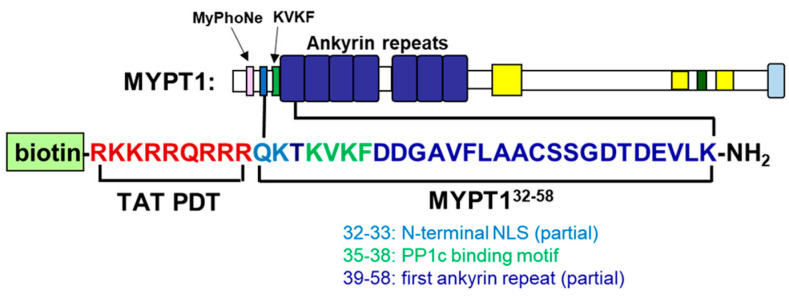
Schematic structure of MYPT1 and amino acid sequence of biotin-TAT-MYPT1^32–58^ peptide.

The peptide to disrupt the PP1c-MYPT1 complex was designed based on the structural data gathered for the subunit interactions by surface plasmon resonance (SPR) studies with MYPT1 fragments and PP1c [6] and by the crystal structure of the PP1c-MYPT1^1–299^ complex [15]. Figure 1 shows the scheme of MYPT1 indicating the major interactive residues/regions with PP1c. These are the MyPhoNE motif, the consensus-binding motif (^35^KVKF^38^) and residues in the ankyrin repeats. The peptide segment N-terminal to the binding motif (including MyPhoNE) was shown to stimulate the myosin phosphatase activity of PP1c [6]. As the goal of our studies was to plan a peptide that would inhibit myosin phosphatase activity by disrupting subunit interactions, this activator peptide segment was not included in the synthesized peptide. Therefore, we chose the sequence from ^32^Q to ^58^K of MYPT1, and this peptide, including the short consensus binding sequence complemented with key residues (A42, V43 and E55) from the first ankyrin repeat, proved to be important in the interaction of MYPT1^1–299^ with PP1c according to the crystal structure of PP1c-MYPT1^1–299^ [15]. We also assumed that this sequence C-terminal to the binding motif might also provide at least partial specificity toward the disruption of PP1-MYPT1 interaction among other PP1-regulatory protein complexes. The peptide was synthesized with the N-terminal addition of biotin and a cell-penetrating transactivating transcriptional activator (TAT) sequence and termed biotin-TAT-MYPT1 as well as biotin-TAT was used as control.

### 3.2. Effect of Biotin-TAT and Biotin-TAT-MYPT1 on the Activity of Myosin Phosphatase Holoenzyme

**Figure 2:**
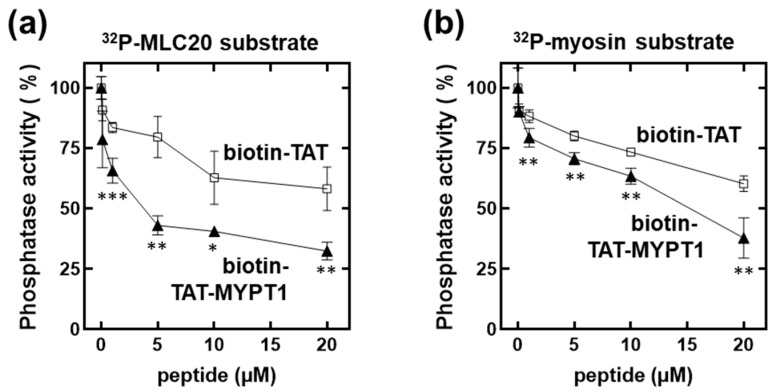
Effects of biotin-TAT/TAT-MYPT1 on the activity of myosin phosphatase holoenzyme. Native myosin phosphatase holoenzyme was assayed in the presence of biotin-TAT (□) or biotin-TAT-MYPT1 (▲) peptide with ^32^P-MLC20 (**a**) or ^32^P-myosin (**b**) substrates as described in Materials and Methods. Values are mean ± SD (*n* = 4). Differences between biotin-TAT and biotin-TAT-MYPT1 effects were analyzed by unpaired two-tailed *t*-test, *p* < 0.05 (*), *p* < 0.01 (**) and *p* < 0.001 (***).

First, we tested the influence of biotin-TAT and biotin-TAT-MYPT1 on the activity of MP holoenzyme using ^32^P-MLC20 (Figure 2a) or ^32^P-myosin (Figure 2b) as substrates. It is apparent that both biotin-TAT and biotin-TAT-MYPT1 suppressed the activity of MP; however, the inhibition caused by biotin-TAT-MYPT1 was significantly more effective than that of biotin-TAT. The inhibitory effect exerted by biotin-TAT is intriguing, but we hypothesized that it occurred by a different mechanism compared to biotin-TAT-MYPT1 and presumably due to basic residues present in the cell-penetrating sequence. In accord with this assumption, it was shown in earlier studies that molecules of basic (polycationic) nature such as histones and protamine as well as polylysine inhibited the activity of PP1-type catalytic subunits [19] and holoenzymes [20]. We considered the application of another protein-based cell-penetrating peptide, penetratin (RQIKIWFQNRRMKWKK) [21], which is somewhat less basic. However, its use was neglected because this peptide included an RVxF motif (IWF) which supposedly binds to PP1c, and, therefore, may not be an appropriate control in these experiments.

### 3.3. Binding of Biotin-TAT and Biotin-TAT-MYPT1 to PP1c

**Figure 3:**
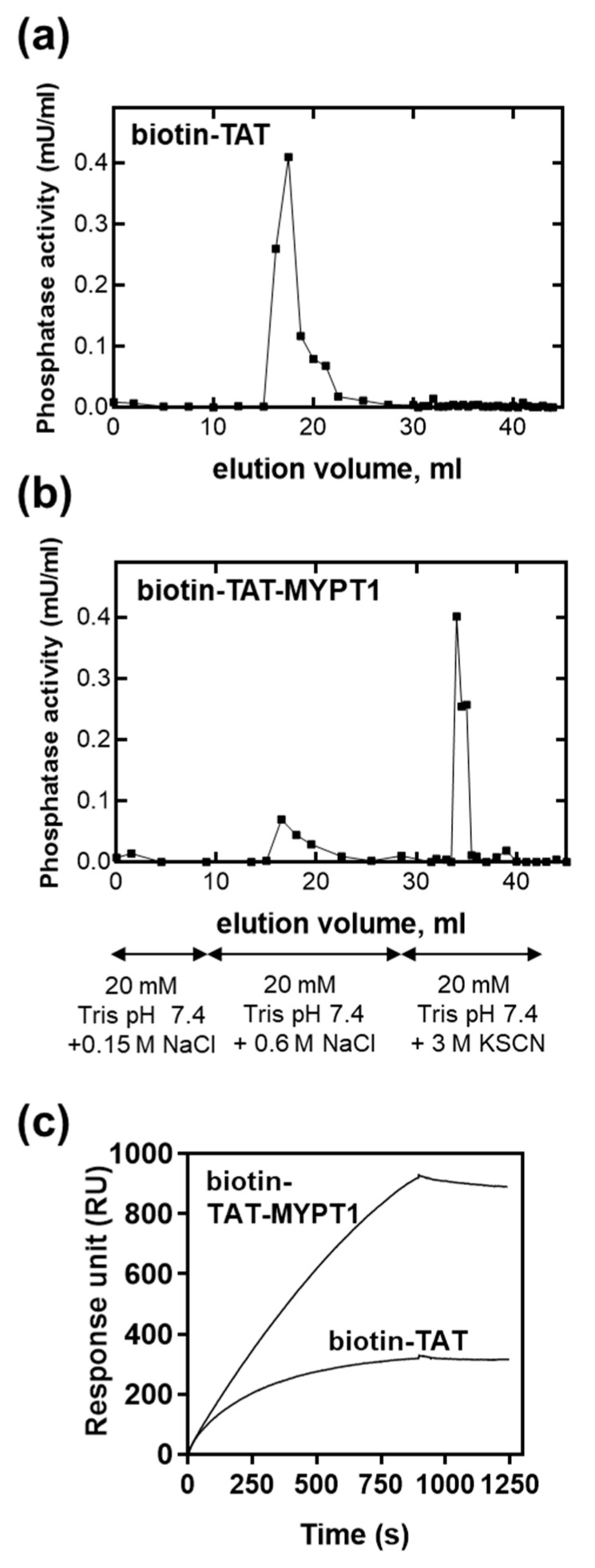
Binding of biotin-TAT/TAT-MYPT1 to PP1cδ. Biotinylated TAT (**a**) or TAT-MYPT1 (**b**) was immobilized on Streptavidin-Sepharose and then purified PP1c was applied. The column was washed with buffers indicated on the graph and eluted fractions were assayed for phosphatase activity. In (**c**), 0.1 µM PP1cδ was injected over the surface of sensor chip SA previously captured with biotin-TAT/TAT-MYPT1. Binding of PP1cδ was monitored as a sensorgram where response unit (RU) values were plotted against time.

Binding of biotin-TAT and biotin-TAT-MYPT1 to PP1c was tested by affinity chromatography and in SPR experiments (Figure 3). Biotin-TAT (Figure 3a) or biotin-TAT-MYPT1 (Figure 3b) were immobilized on Streptavidin Sepharose beads, and then purified PP1c was flowed through the column in a running buffer. PP1c bound to both matrixes, and in the case of the immobilized biotin-TAT, it was eluted by 0.6 M NaCl (Figure 3a). In contrast, from the biotin-TAT-MYPT1-coupled matrix, a very low amount of PP1c was eluted by 0.6 M NaCl, but it was able to be recovered from the column by applying 3 M KSCN for elution (Figure 3b). As this high concentration of chaotropic salt was needed to elute PP1c from an immobilized MYPT1^1–296^ column it was concluded that biotin-TAT-MYPT1 formed a similarly stable interaction with PP1c to that identified with the N-terminal 296 residues of MYPT1 [22]. Moreover, the low amount of PP1c eluted by 0.6 M NaCl from the biotin-TAT-MYPT1-coupled column implies that TAT did not significantly interfere with the binding of MYPT1^32–58^ peptide to PP1c; therefore, the major interactions in the PP1c-biotin-TAT-MYPT1 complex occurred via MYPT1^32–58^.

Pull-down assays with Streptavidin coupled biotin-TAT or biotin-TAT-MYPT1 from A7r5 cell lysates were carried out and the results are presented as a supplementary figure (Figure S1). It shows that both Streptavidin coupled biotin-TAT and biotin-TAT-MYPT1 pulled-down PP1c quantitatively from cell lysate leaving no PP1c in the unbound fraction. In addition, washing the precipitate with buffers containing 0.6 M NaCl or 3 M KSCN also proved the different binding strength of PP1c with biotin-TAT and biotin-TAT-MYPT1.

Biotin-TAT or biotin-TAT-MYPT1 was also immobilized on Streptavidin coupled (SA) sensor chips and binding of PP1c to these sensor chip surfaces was recorded (Figure 3c). This experiment confirmed that PP1c bound to biotin-TAT-MYPT1 with higher preference and association kinetics than to biotin-TAT (k_on_ = 5.79 × 10^3^ s^−1^ for biotin-TAT-PP1c or k_on_ = 1.74 × 10^4^ s^−1^ for biotin-TAT-MYPT1-PP1c complex formation, respectively), also indicating a different mechanism of binding in the case of these two interacting molecules.

**Figure 4:**
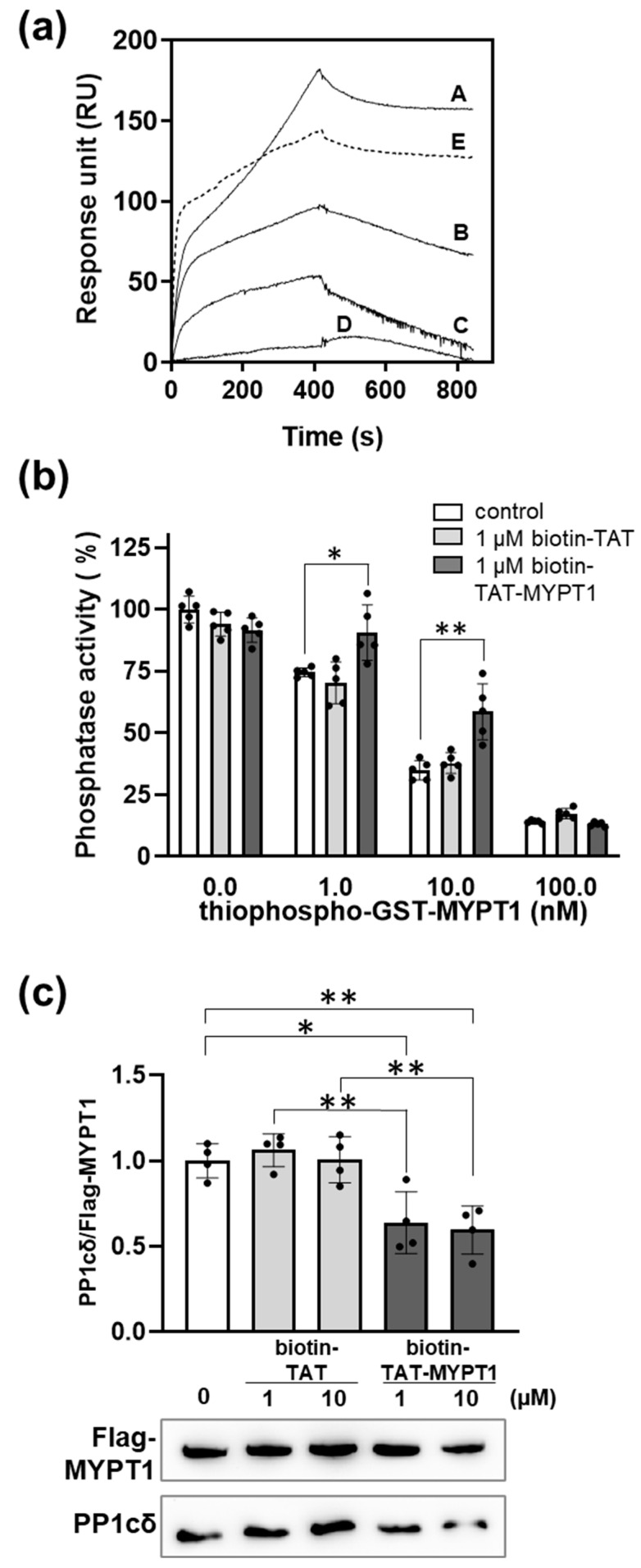
The effect of biotin-TAT/TAT-MYPT1 on subunit interaction of myosin phosphatase. (**a**) GST-MYPT1 was immobilized on anti-GST antibody-coupled sensor chip, then 3 µM PP1cδ was injected over the surface in the presence of 0 (A), 1 (B), 5 (C) and 10 (D) µM biotin-TAT-MYPT1 or 10 µM biotin-TAT (E). Sensorgrams were obtained using Biacore 3000 instrument as described in Materials and Methods. (**b**) Purified PP1c was pre-incubated with none (control) or 1 µM biotin-TAT/TAT-MYPT1 and then with 0–100 nM thiophosphorylated GST-MYPT1. Phosphatase activity was measured with ^32^P-MLC20 and plotted as a percentage of control. Values are means ± SD (*n* = 5). (**c**) Flag-MYPT1-PP1cδ complex isolated on anti-Flag affinity resin was incubated with 0, 1 or 10 µM biotin-TAT/TAT-MYPT1 and resin-bound proteins were analyzed by Western blot experiment. PP1cδ band intensities were normalized for Flag-MYPT1 and expressed relative to the control. Values are means with SD (*n* = 4). Differences between groups were analyzed by unpaired two-tailed *t*-test, *p* < 0.05 (*), *p* < 0.01 (**).

Further experiments were carried out for the interaction of biotin-TAT-MYPT1 and PP1c with respect to how it influenced the association of holoenzyme formation of PP1c with MYPT1. Recombinant GST-MYPT1 was immobilized on anti-GST-coupled sensor chip and binding of PP1c was recorded in SPR experiments (Figure 4a). As illustrated in Figure 4a, biotin-TAT-MYPT1 suppressed binding of PP1c to GST-MYPT1 in a concentration-dependent manner indicating competition between MYPT1 and biotin-TAT-MYPT1 for binding to PP1c. In contrast, biotin-TAT decreased binding of PP1c to GST-MYPT1 only slightly at 10 µM concentration at which biotin-TAT-MYPT1 suppressed binding almost completely. Figure 4b implicates that biotin-TAT-MYPT1 competed with binding of thiophosphorylated MYPT1 (on Thr696 and Thr853) to PP1c in a phosphatase assay and partially relieved the inhibition caused by this inhibitory MYPT1 form. In contrast, biotin-TAT was without effect on the inhibition of PP1c by thiophosphorylated MYPT1. The peptides were also assayed on their ability to dissociate the PP1c-MYPT1 complex. Flag-MYPT1 was overexpressed in tsA201 cells, then immunoprecipitated with anti-Flag resin and the immunoprecipitate was incubated in the absence or presence of different concentration of biotin-TAT or biotin-TAT-MYPT1. The resin was washed and then analyzed for Flag-MYPT1 and PP1cδ by Western blotting (Figure 4c). As shown in Figure 4c, in the presence of biotin-TAT-MYPT1, significantly less PP1c remained associated with Flag-MYPT1 compared to its absence. These data indicate that biotin-TAT-MYPT1 induced dissociation of the Flag-MYPT1-PP1c complex, while biotin-TAT was without effect. These latter two experiments (Figure 4b,c) provided further evidence for the distinct binding mechanisms of biotin-TAT to PP1c in contrast to biotin-TAT-MYPT1 peptide. Biotin-TAT did not dissociate the MP holoenzyme and had no effect on phosphatase activity in the presence of thiophosphorylated MYPT1. On the other hand, it is apparent that biotin-TAT-MYPT1 not only competed with MYPT1 for binding but even dissociated PP1c from the immunoprecipitated holoenzyme formed under cellular conditions.

### 3.4. Effect of Biotin-TAT-MYPT1 on the Phosphatase Activity in A7r5 Cells

**Figure 5:**
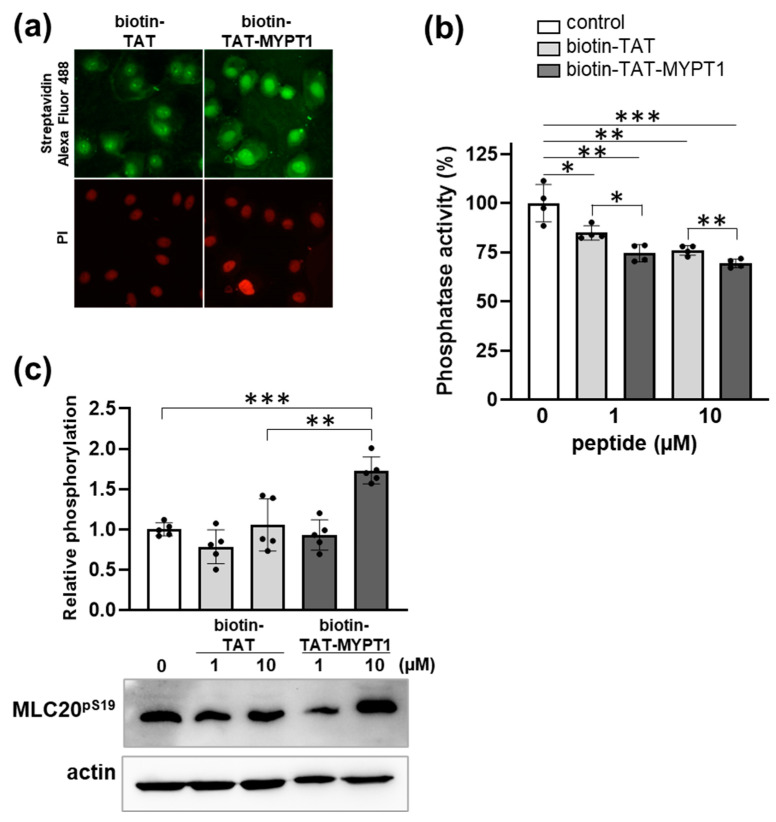
Effect of biotin-TAT-MYPT1 on phosphatase activity in A7r5 cells. (**a**) A7r5 cells were transduced with 1 µM biotin-TAT or biotin-TAT-MYPT1 for 30 min. Cells were stained with Alexa Fluor 488-conjugated Streptavidin and propidium iodide (PI) to visualize biotinylated peptides and cell nucleus, respectively. (**b**) A7r5 cells were treated with none, 1 or 10 µM biotin-TAT/TAT-MYPT1 for 30 min. Phosphatase activity of cell lysates was determined using ^32^P-myosin substrate and plotted as a percentage of control. Values are means ± SD (*n* = 4). (**c**) Representative Western blot of biotin-TAT/TAT-MYPT1-treated A7r5 cells using anti-MLC20^pS19^ and anti-actin antibodies. Band intensities were normalized to actin and expressed relative to the untreated control. Values are means with SD (*n* = 5). Differences between groups were analyzed by unpaired two-tailed *t*-test, *p* < 0.05 (*), *p* < 0.01 (**) and *p* < 0.001 (***).

The cell-penetrating ability of biotin-TAT and biotin-TAT-MYPT1 peptides and their influence on cellular phosphatase activity were probed using A7r5 smooth muscle cell line. The biotin tag in the peptides made it possible to assess the localization of the peptides within the cells with fluorescent microscopy by staining with Alexa Fluor 488-conjugated Streptavidin. Figure 5a indicates that both biotin-TAT and biotin-TAT-MYPT1 penetrated into A7r5 cells and localized in the cytoplasm and the nucleus. Assaying A7r5 cell lysates for phosphatase activity with ^32^P-myosin substrate showed inhibition by both peptides in a concentration dependent manner; however, biotin-TAT-MYPT1 exerted moderately, but significantly, higher suppressive effect on dephosphorylation compared to biotin-TAT (Figure 5b). Next, the phosphorylation level of the cellular substrate (P-MLC20) was also assessed using anti-MLC20^pS19^ antibody for Western blotting of cell lysate. It is apparent that biotin-TAT did not influence MLC20^pS19^ level even when applied in 10 µM, while biotin-TAT-MYPT1 enhanced the MLC20^pS19^ level significantly at the same concentration. These data are consistent with the conclusion that the biotin-TAT-MYPT1 peptide penetrates cells and inhibits MP activity, presumably via dissociating PP1c-MYPT1 interaction, thereby counteracting with the substrate targeting effect of MYPT1. It has been assumed that under cellular conditions, P-myosin is dephosphorylated effectively only by MP. The inhibition of P-myosin dephosphorylation by biotin-TAT-MYPT1 might indicate a specific effect of this disrupting peptide on MP.

## 4. Conclusions

The PP1 family of P-Ser/Thr specific protein phosphatases has been shown to be regulated by short cell-penetrating peptides including the RVxF PP1c-binding motif [11,12,13,14,23] and the current hypothesis is that these peptides disrupt interaction between PP1c and its regulator/inhibitor molecules. In the present study, we planned a longer peptide in which the short sequence including the RVxF binding motif was supplemented with another PP1c interactive region from the ankyrin repeat of MYPT1 (biotin-TAT-MYPT1) to increase the specificity toward the disrupting action exerted on the PP1c-MYPT1 interaction in the myosin phosphatase (MP) holoenzyme. This peptide penetrated into cells, inhibited myosin phosphatase activity by presumably dissociating the PP1c-MYPT1 complex and increased cellular myosin phosphorylation. Localization of the peptide within the cell established that biotin-TAT-MYPT1 predominantly accumulated in the nucleus and was relatively scarce in the cytoplasm. We do not know the exact extent of the penetration and the distribution of biotin-TAT-MYPT1 within the cells; however, it is noteworthy that even the presumably low concentration of the peptide in the cytoplasm resulted in significant inhibition of dephosphorylation of myosin, a cytoskeletal substrate. It is of note that MP, in spite of its specific name, also dephosphorylates other cytoskeletal proteins as well as regulates numerous cellular processes acting on novel substrates [24]. Future directions of the studies could be to assess the effect of biotin-TAT-MYPT1 on these physiological processes putting special emphasis to reveal its nuclear influences. We consider biotin-TAT-MYPT1 as an initial template for development of future MP inhibitory molecules, even with pharmacological relevancies. Firstly, the TAT-peptide may be replaced by other penetrating sequences to increase cytoplasmic localization of the MYPT1^32–58^ peptide segment, as the TAT-peptide seems to be responsible for the predominant nuclear localization. In addition, the biotin-TAT peptide itself inhibits phosphatase activity, although our results suggest that it occurs by less stable interaction with PP1c and a different binding mechanism from that of biotin-TAT-MYPT1. Secondly, cellular stability of the peptide (especially against proteolytic attack) may be increased by using D-amino acids for the synthesis in “retroinverso” manner. It was shown earlier that such peptides also exerted the expected disrupting efficacy [14,23] on PP1 and its binding partners. Nevertheless, in our present study, the peptide tailored by the “native” MYPT1 sequence was effective on cells after a relatively short incubation time, but its intracellular lifetime remained elusive.

In summary, we have introduced a novel peptide to disrupt interaction between the two major binding subunits of MP and hypothesized at least its partial specificity toward this holoenzyme. This peptide may serve as template to develop a more effective molecule even with higher specificity toward MP and longer intracellular lifetime with possible pharmacological applications.

## Data Availability

The original contributions presented in this study are included in the article/Supplementary Materials. Further inquiries can be directed to the corresponding authors.

## Supplementary Materials

The following supporting information can be downloaded at: https://www.mdpi.com/article/10.3390/biom15050705/s1, Figure S1: Pull-down assays with Streptavidin coupled biotin-TAT or biotin-TAT-MYPT1 from A7r5 cell lysates. Original Western Blots images

## Abbreviations

The following abbreviations are used in this manuscript:

biotin-TAT	biotinylated TAT peptide
biotin-TAT-MYPT1	biotinylated TAT peptide coupled with MYPT1^32–58^ fragment
MLC20	20 kDa light chain of myosin
MP	myosin phosphatase
MYPT1	myosin phosphatase target subunit-1
PP1	protein phosphatase-1
PP1c	PP1 catalytic subunit
SPR	surface plasmon resonance
TAT	HIV-derived cell-penetrating peptide (transactivating transcriptional activator)
