# Supplementary material for: Cell-Penetrating Peptide Based on Myosin Phosphatase Target Subunit Sequence Mediates Myosin Phosphatase Activity
Source: Biomolecules. 2025 May 12;15(5):705. doi: 10.3390/biom15050705 (PMC12110079; doi:10.3390/biom15050705)
Supplement: Supplementary file 1 [file biomolecules-15-00705-s001.zip › biomolecules-3531309-original figures.docx]

# Figure 4.C

biotin- TAT

biotin- TAT- MYPT1

X 0 1 10 1 10 (µM)

kDa


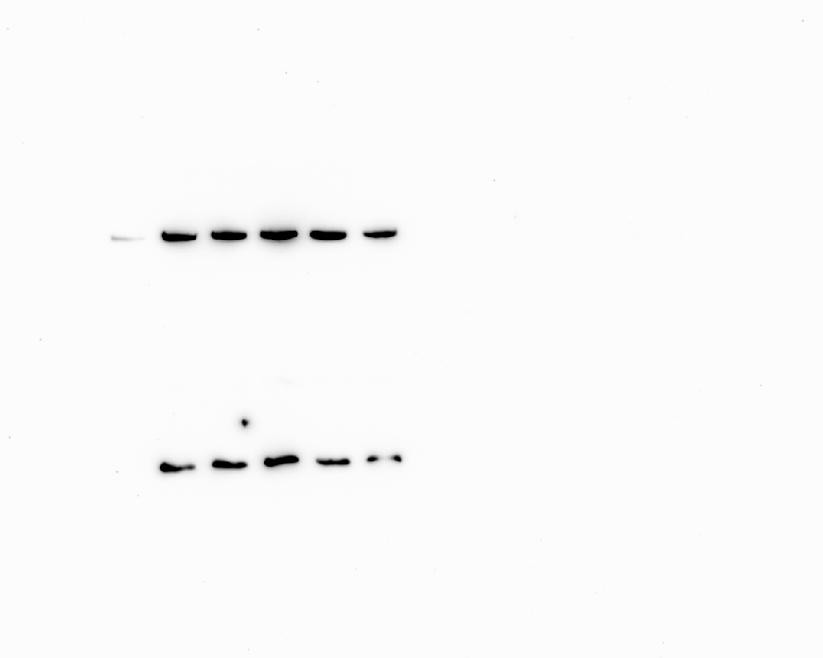


250

150

100

75

50

37

**Flag-**

**MYPT1**

**PP1cδ**

|  | | | **biotin-TAT** | | **biotin-TAT-MYPT1** | |
| --- | --- | --- | --- | --- | --- | --- |
|  | µM | 0 | 1 | 10 | 1 | 10 |
| intensity ratios | **Flag-MYPT1** | 1.00 | 1.16 | 1.40 | 1.29 | 0.64 |
|  | **PP1cδ** | 1.00 | 1.16 | 1.47 | 0.76 | 0.46 |

# Figure 5.A

**
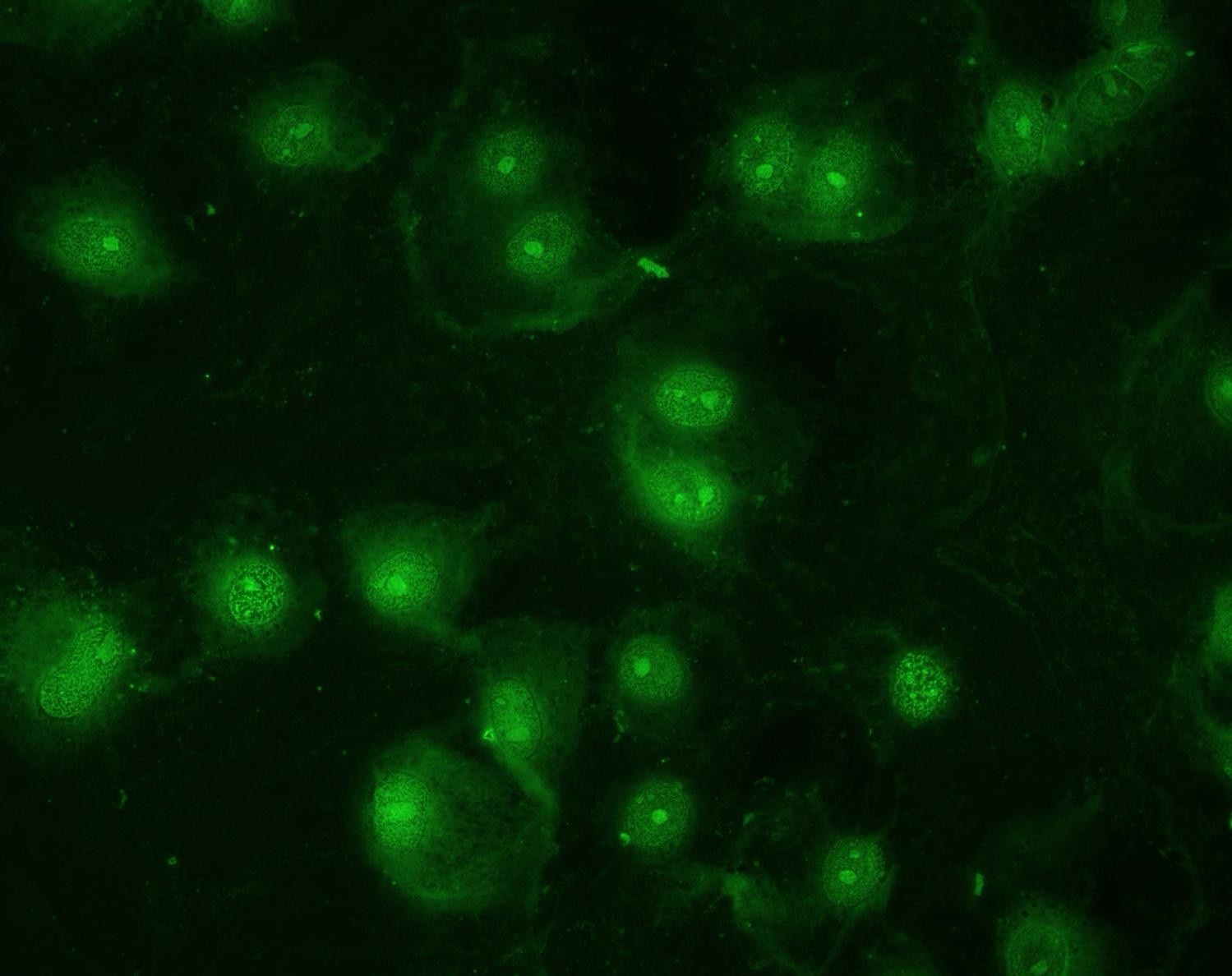
biotin- TAT**

**Streptavidin, Alexa Fluor 488**

# Figure 5.A

**biotin-**

**
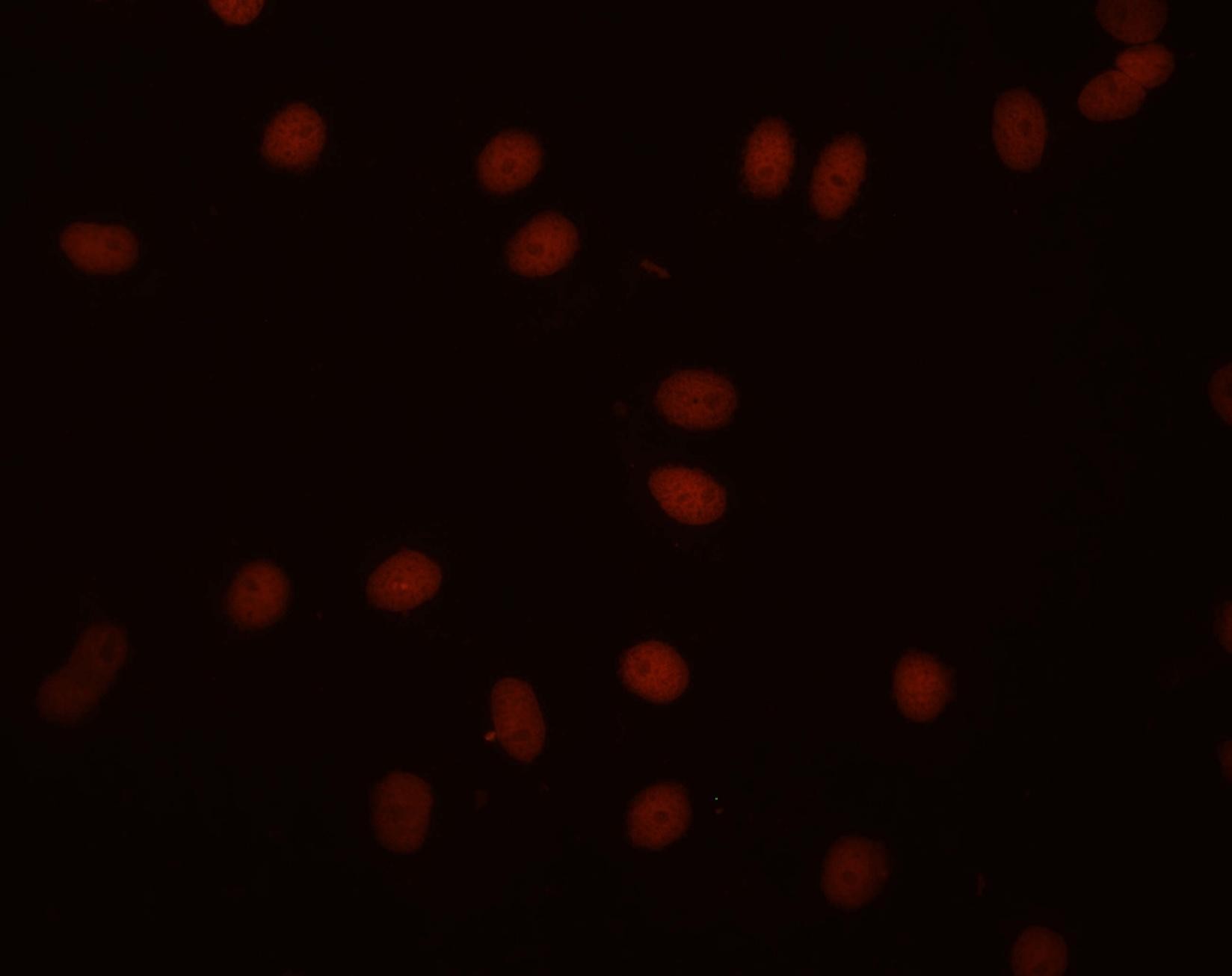
TAT PI**

# Figure 5.A

**
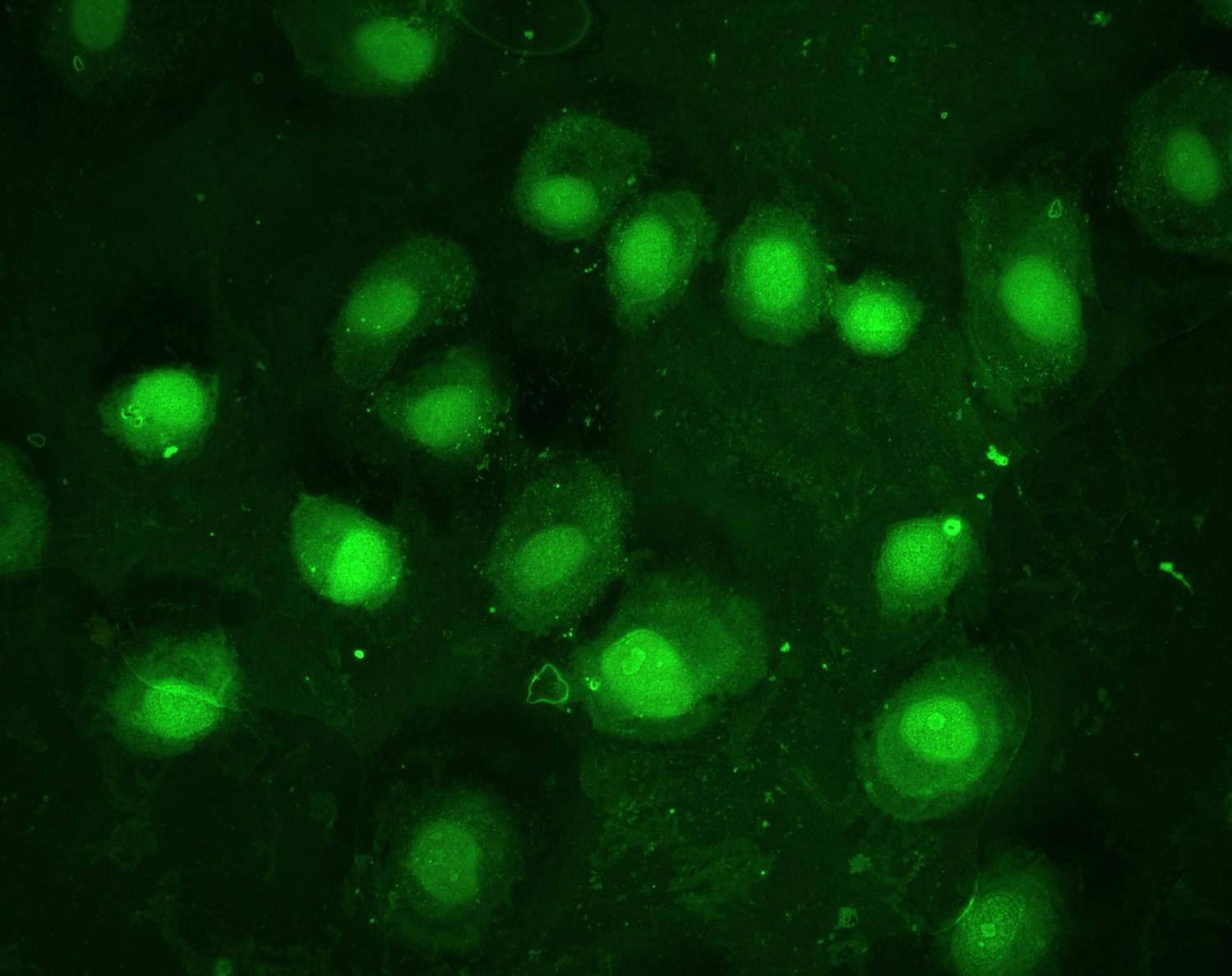
biotin- TAT-MYPT1**

**Streptavidin, Alexa Fluor 488**

# Figure 5.A

**biotin- PI**

**
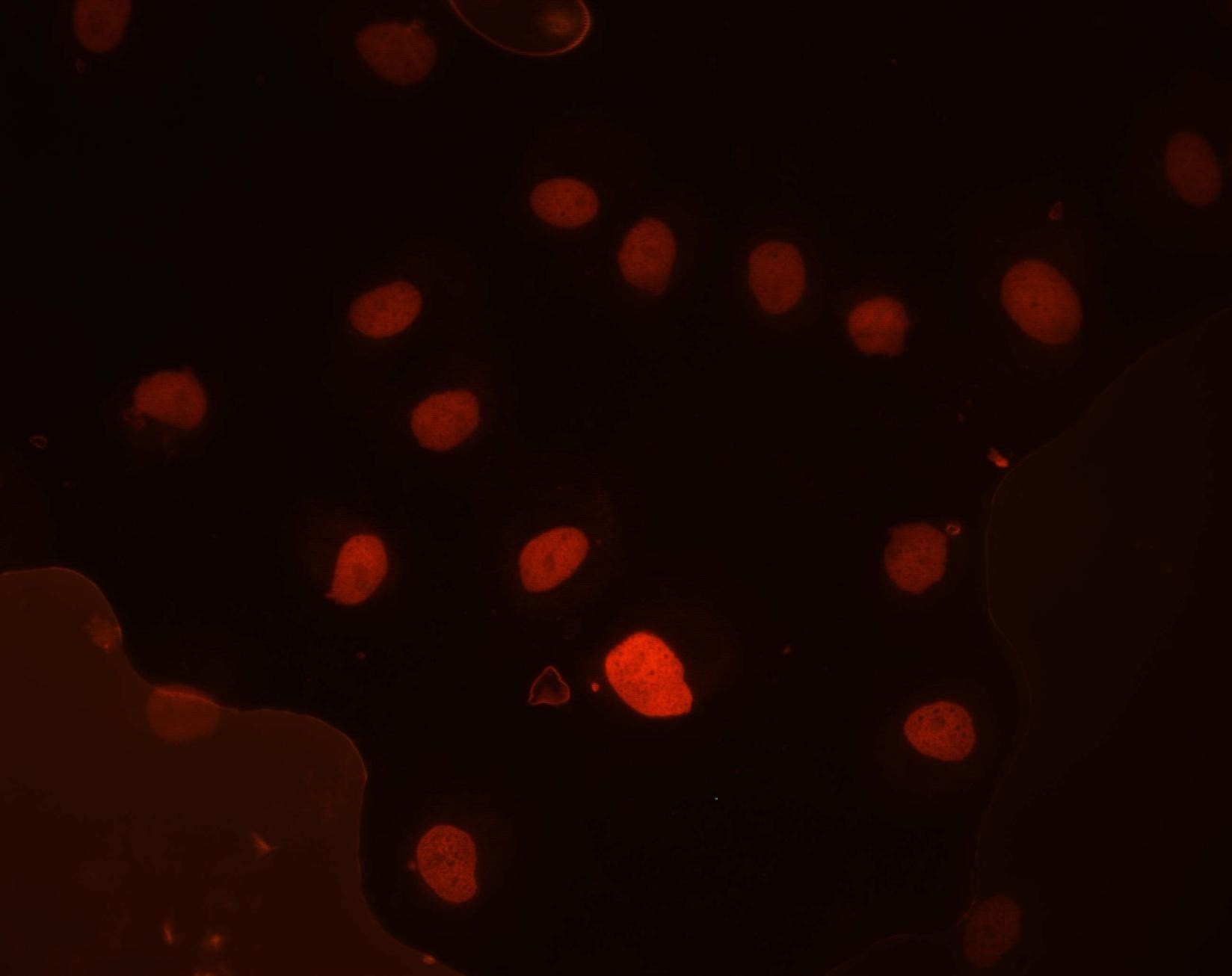
TAT-MYPT1**

# Figure 5.C

biotin-

TAT

biotin- TAT-MYPT1

0 1 10 1 10

(µM)

kDa 25


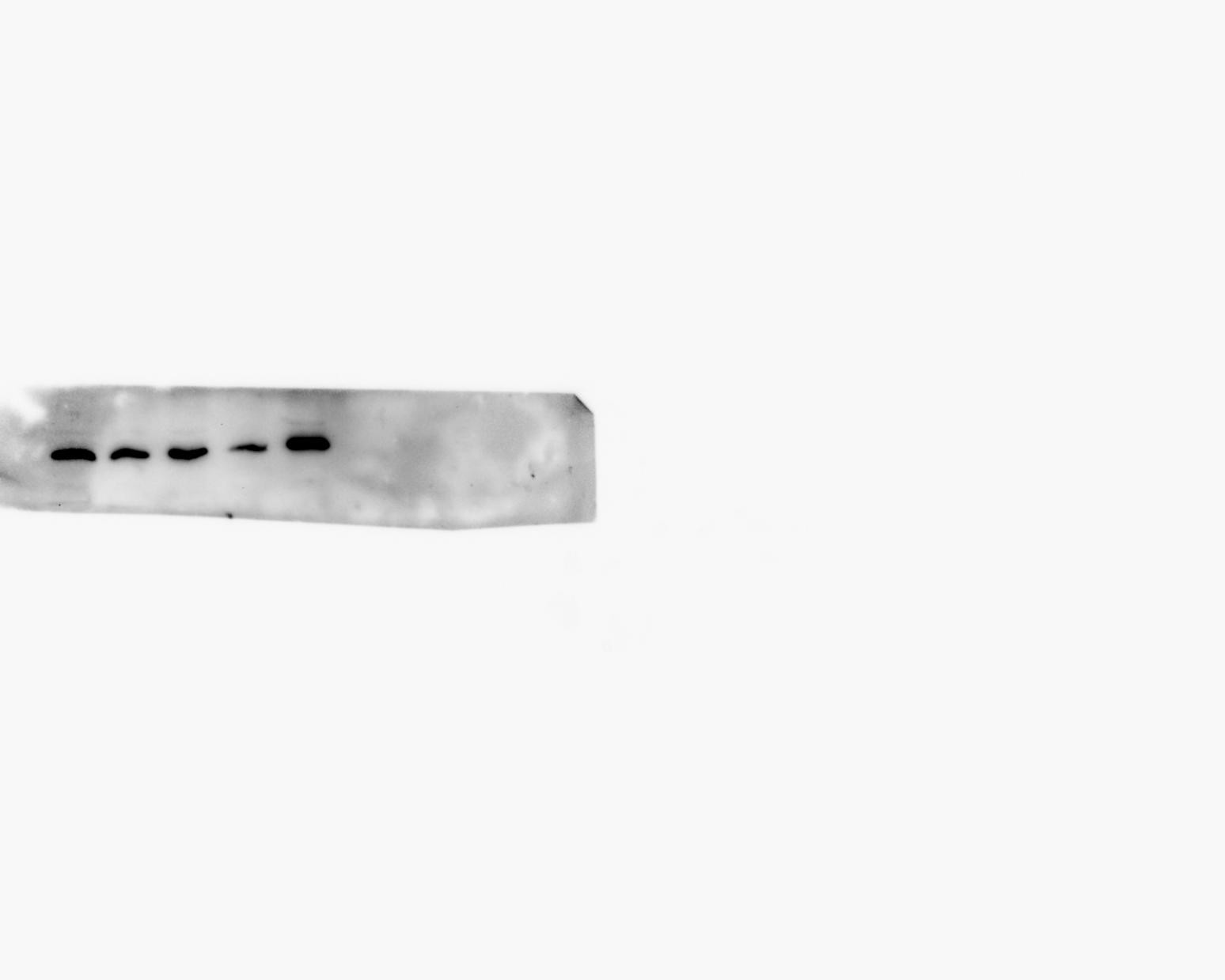


20 15

**Anti-MLC20pS19**

|  | | | biotin-TAT | | biotin-TAT-MYPT1 | |
| --- | --- | --- | --- | --- | --- | --- |
|  | µM | 0 | 1 | 10 | 1 | 10 |
| intensity  ratios | P-MLC20 | 1.00 | 0.51 | 0.68 | 0.31 | 1.22 |
|  | actin | 1.00 | 0.97 | 1.12 | 0.73 | 0.69 |

# Figure 5.C

biotin-

TAT

biotin- TAT-MYPT1

0 1 10 1 10

(µM)

kDa


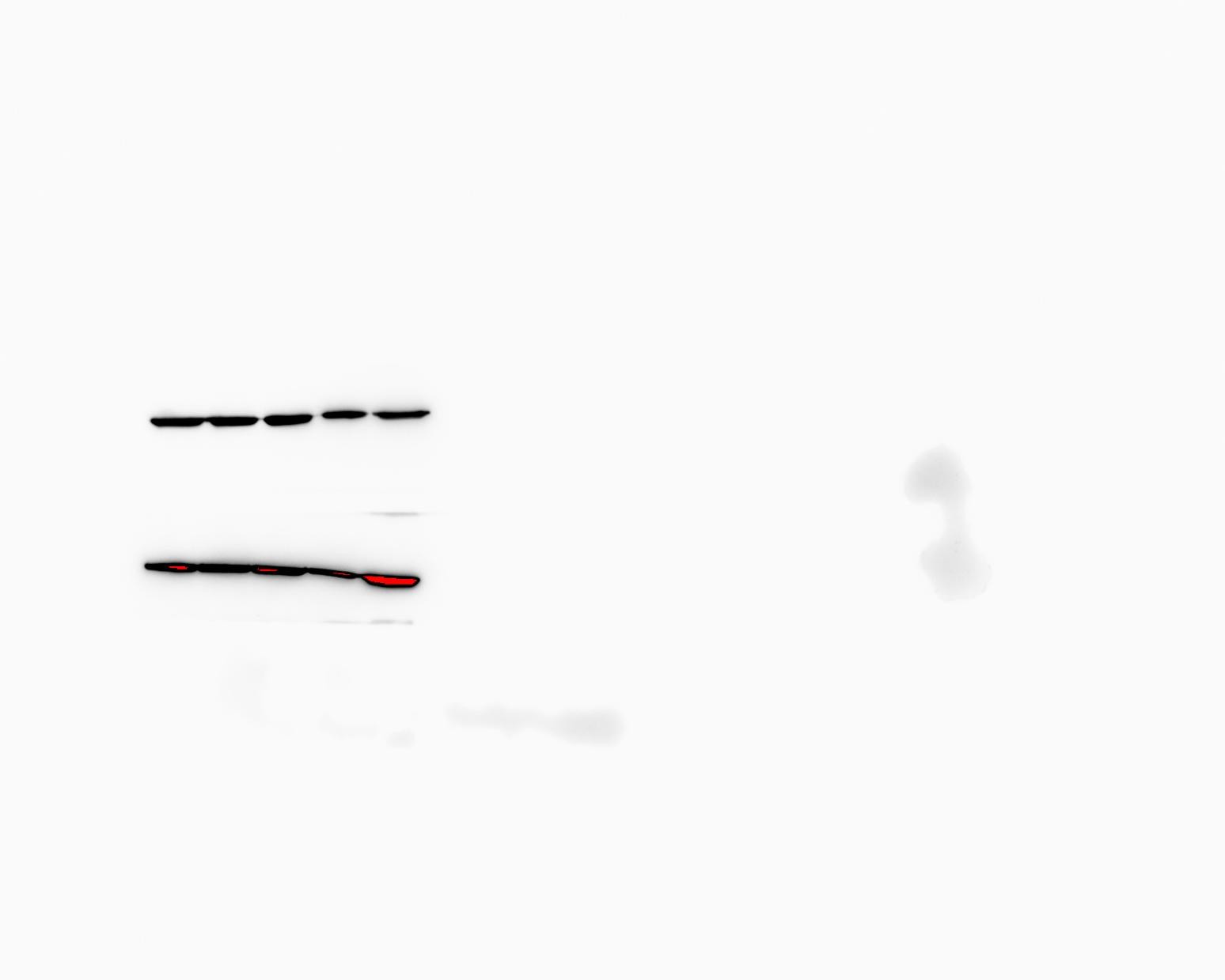


50

37

**Not relevant**

**Anti-actin**

|  | | | biotin-TAT | | biotin-TAT-MYPT1 | |
| --- | --- | --- | --- | --- | --- | --- |
|  | µM | 0 | 1 | 10 | 1 | 10 |
| intensity  ratios | P-MLC20 | 1.00 | 0.51 | 0.68 | 0.31 | 1.22 |
|  | actin | 1.00 | 0.97 | 1.12 | 0.73 | 0.69 |

#
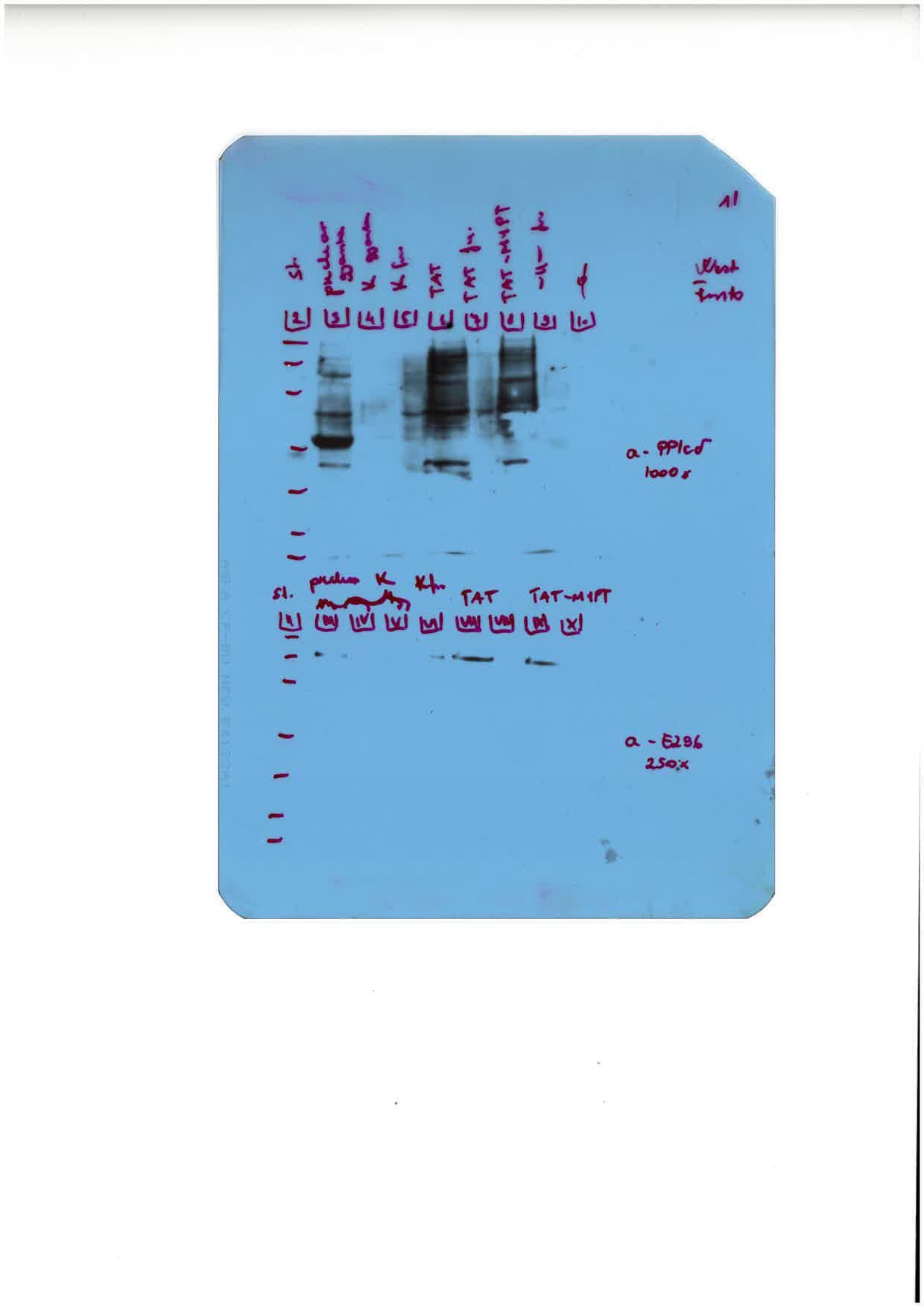
Figure S1. (a)

**Figure S1. (a)**

**Streptavidin**

**+ biotin-**

**+ biotin-**

**Sepharose**

**input**

**TAT**

**TAT-MYPT1**

**
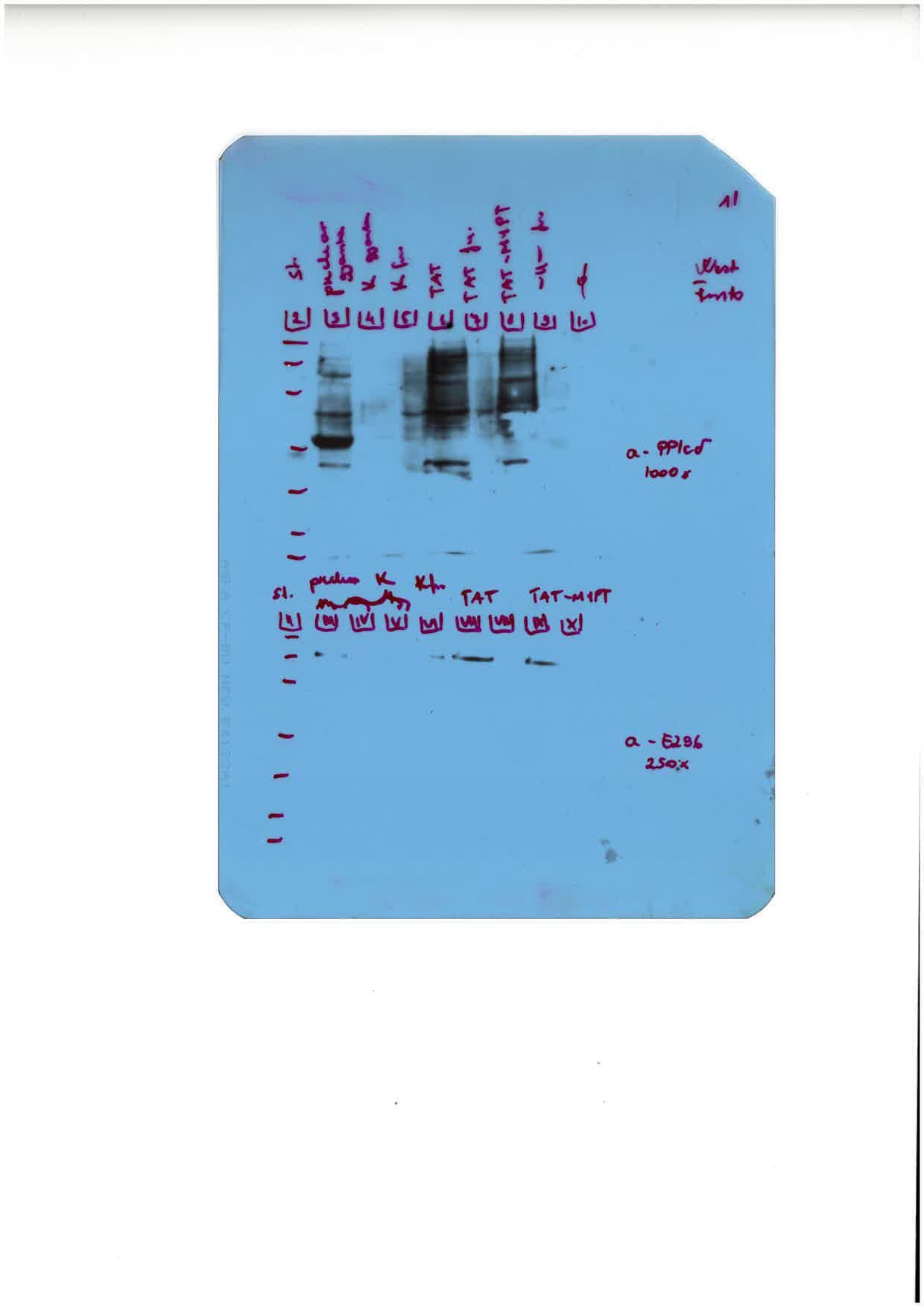
bead ub bead ub bead ub X**

**Not**

**relevant**

**anti- PP1cδ**

**Not relevant**

#
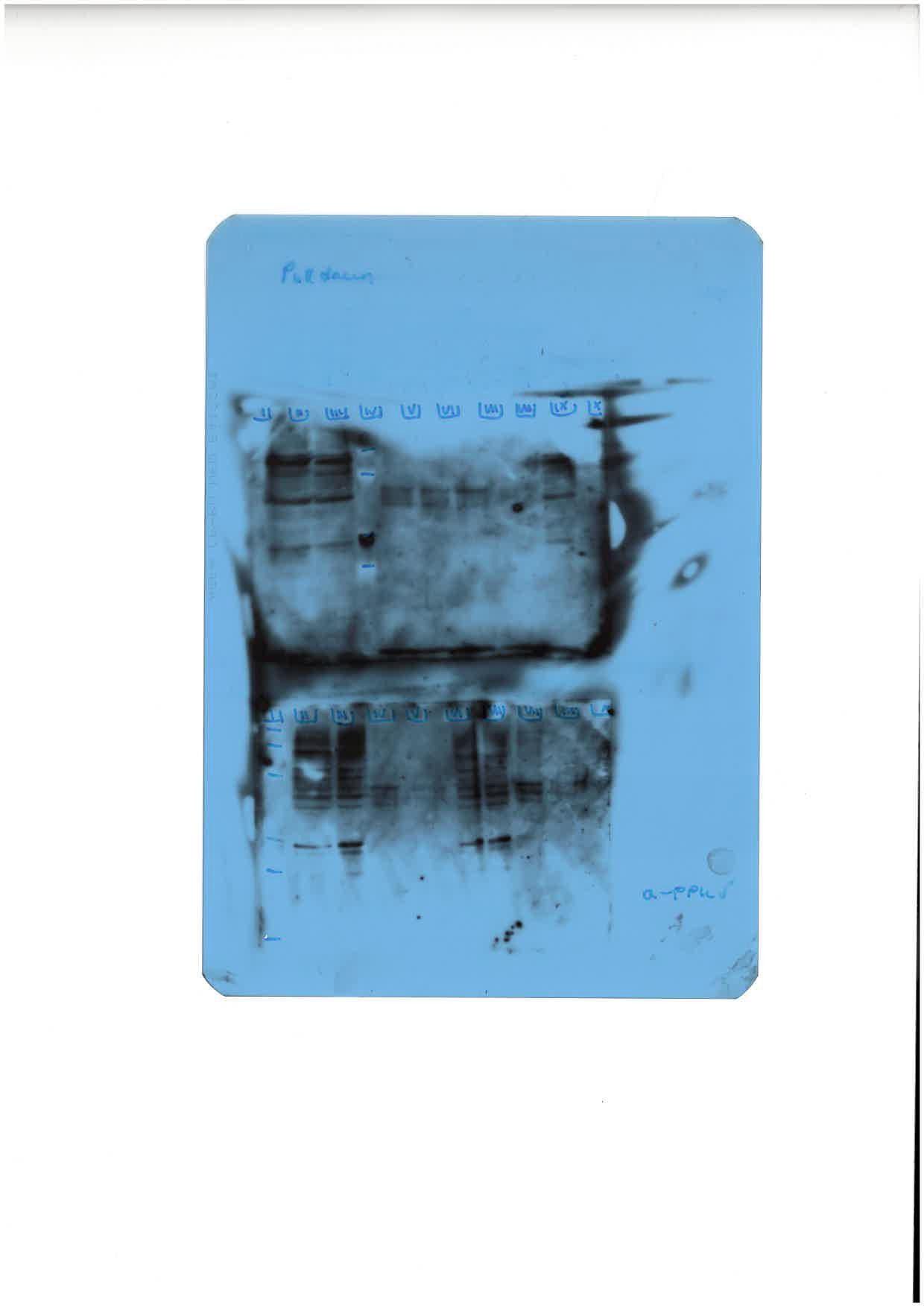
Figure S1.B

**Figure S1.B**

**Not relevant**

**Not relevant**

**anti- PP1cδ**

X

| **0.15 M NaCl** | **- +** | **+** | **+** | **+** | **+** | **+** | **X** |
| --- | --- | --- | --- | --- | --- | --- | --- |
| **0.6 M NaCl** | **- -** | **+** | **+** | **-** | **+** | **+** |  |
| **3 M KSCN** | **- -** | **-** | **+** | **-** | **-** | **+** |  |

**input**

**biotin-TAT**

**biotin-TAT-MYPT1**


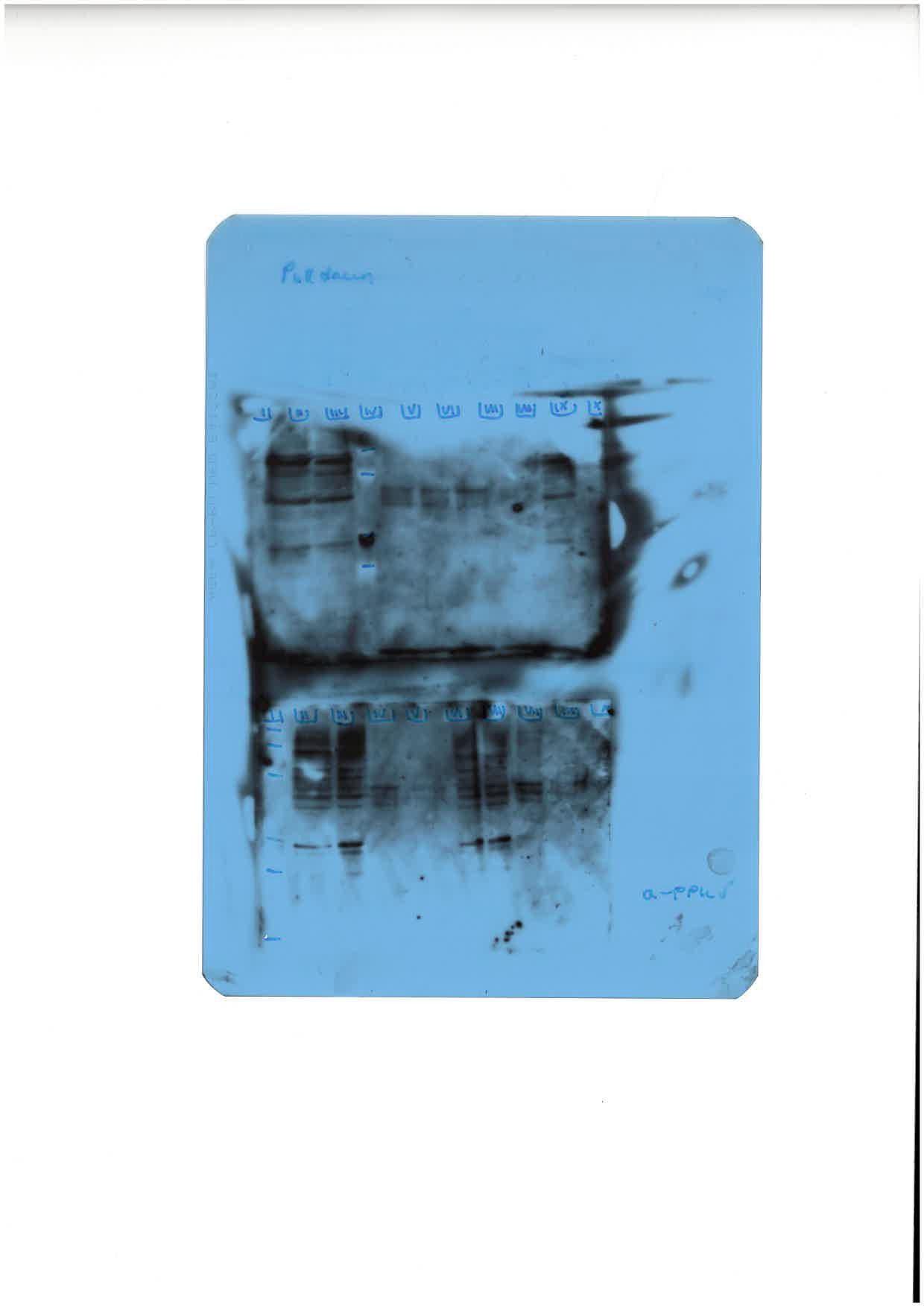


**Streptavidin Sepharose**
