# Supplementary material for: Cell-Penetrating Peptide Based on Myosin Phosphatase Target Subunit Sequence Mediates Myosin Phosphatase Activity
Source: Biomolecules. 2025 May 12;15(5):705. doi: 10.3390/biom15050705 (PMC12110079; doi:10.3390/biom15050705)
Supplement: Supplementary file 1 [file biomolecules-15-00705-s001.zip › biomolecules-3531309-supplementary.pdf]

Figure S1.

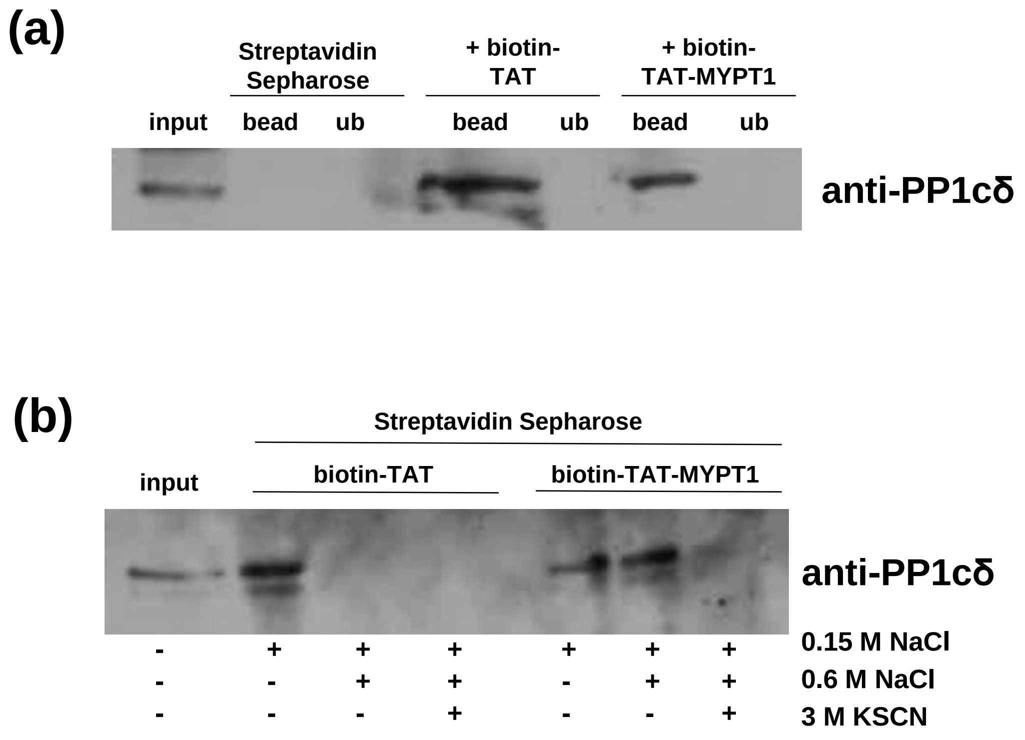

(a) Pull-down experiments using uncoupled or biotin-TAT/TAT-MYPT1-coupled Streptavidin Sepharose and A7r5 cell lysate was performed as described in Materials and methods. Resin-bound (bead) and unbound (ub) samples were analyzed for the presence of PP1cδ by Western blotting. **(b)** Pull-down experiments were performed as above with additional washing steps. Samples were taken from the beads and analyzed by Western blotting as previously.
